# Special needs of refugees with substance use disorders from conflict-affected countries: a comparison with German-born individuals

**DOI:** 10.1007/s00127-025-02842-2

**Published:** 2025-02-25

**Authors:** Mayte López-Atanes, Moritz Rosenkranz, Leire Erkoreka, Maria Recio-Barbero, Melav Bari, Philipp Hiller, Simone Penka, Jutta Lindert, Marcus Martens, Ingo Schäfer

**Affiliations:** 1 Center for Interdisciplinary Addiction Research, University of Hamburg, Hamburg, Germany; 2 Department of Psychiatry and Psychotherapy, University Medical Center Hamburg-Eppendorf, Hamburg, Germany; 3 University of the Basque Country, Leioa, Spain; 4 Charité University Medicine, Berlin, Germany; 5 University of Applied Sciences Emden/Leer, Emden, Germany

**Keywords:** Addiction, Substance use disorders, Refugees, Asylum seekers, Health services

## Abstract

**Background:**

Refugees seem to be at enhanced risk for substance use disorders. At the same time, they have less access to services and their health needs remain poorly understood. This study aims to evaluate the specific needs of refugees as compared to German-born individuals seeking help for substance use disorders.

**Methods:**

We conducted an observational case–control study using all treatment episodes in outpatient addiction facilities in three German federal states during the year 2020. A total of 719 refugee clients were matched to 713 German-born individuals using propensity score matching. We compared potential needs related to features of substance use as well as different psychosocial areas.

**Results:**

The final sample of refugees was composed of n = 384 (26.8%) cases from Afghanistan, n = 214 (14.9%) from Syria and n = 121 (8.4%) from Iraq. Clients consulted mostly due to cannabis use (44.8%) or opioid use (20.1%). Intravenous use of drugs and needle sharing was significantly lower in refugees (p < 0.05). A higher proportion of refugees than Germans lived in provisional housing, were unemployed or in charge of minor children (p < 0.05). Mental and physical comorbidities were significantly higher in refugees (p < 0.05). They were also more likely to have been victims of violence and less likely to present violent behaviors.

**Conclusion:**

Refugees with substance use disorders differ regarding a spectrum of psychosocial issues from German-born individuals. This highlights the need to link addiction treatment with other parts of the health care and psychosocial support systems to provide adequate care for this group.

## Introduction

In the last decade, Europe received an increasing number of refugees.^1^ Germany has been one of the main destination countries. In 2021 Germany received 27.7% of all refugees in Europe, followed by France and Spain [1]. By the middle of 2021 Germany had admitted more than 1.2 million people, according to UNHCR [2]. People from Syria, Afghanistan, and Iraq represented the three largest groups of asylum seekers – together accounting for almost 40% of all first-time asylum applications in the EU Member States in 2021 [3]. For all three groups, the largest numbers were documented in Germany (accounting 55.8% of all asylum seekers from Syria in the EU, 27.9% of people from Afghanistan, and 60.1% from Iraq). This population consists mainly of young men: about 70% are male, and 80% are less than 35 years old [1].

Refugees often face highly distressing situations, from violence and persecution in their homeland to potentially traumatic experiences during flight and resettlement [4, 5]. In the recipient country, they are often faced with postmigration stressors, like insecure residency status, challenging asylum procedures, restricted access to services, and lack of opportunities to work or study [6, 7]. Both, experiences of violence and postmigration stressors can cause or exacerbate mental health symptoms [8]. Stressful experiences and living conditions can also have a deleterious impact on physical health. For example, prolonged waiting times to obtain a refugee status have been related to a poorer physical condition [7].

It has long been noted that refugees are also at increased risk for substance-related disorders [9–11]. In a more recent review by Horyniak et al. [12] risky alcohol use was found in 17–36% of refugees in camps or shelters, and 4–7% after resettlement in other settings. Being a man, previous psychiatric history as well as traumatic experiences have been described to be the main risk factors [11, 13–16] but other risk factors, also seem to play a role. This has been exemplified by a study among refugees resettled in Melbourne, where alcohol consumption was a common method to deal with trauma, but also with boredom, and frustration [17, 18]. Similar findings have been reported from Finland [19] Other studies further underlined the association between daily stressors and psychiatric outcomes, including substance-related disorders [16, 20, 21]. For example, in Australia, the biggest difficulties of refugee youth with comorbid mental health and substance use disorders were social disconnectedness, as well as lack of employment and education [6].

In general, refugees are less prone to seek help and have less access to the health care system [22]. A scoping review by Pluck et al. [19] reported that monetary, social, language barriers and a lack of knowledge of the health care system in the host country were frequent problems that refugees had to face when trying to get professional help. Refugees with substance use disorders are even less likely to be reached by services [23]. Additional barriers for help-seeking in this group include differing concepts and explanatory models for substance abuse, as well as the stigma related to substance use disorders [24]. There is a clear need for substance abuse services to address such additional barriers and adapt their practice to the needs of multicultural populations [23] but only few studies to date examined the differential needs of refugees with substance use disorders as compared to other groups. Our objective was to compare the clinical profile of refugees with substance use disorders with a German-born group and describe the differences or similarities.

## Materials and methods

### Study design

We conducted cross-sectional study based on data from the German Core Dataset (KDS). The KDS was initiated in 1998 as a common process of data compilation in all addiction treatment centers in Germany [25]. We extracted relevant outpatient clinical data from the federal states of Hamburg, North-Rhine-Westphalia, Hesse and Schleswig–Holstein, covering about 400 treatment facilities. As data is fully anonymized, no ethical consent was required.

### Participants

We selected all treatment episodes that started in the year 2020 (N = 37.509). Data were analyzed in July 2022. Only individuals between 12 and 90 years were included. We excluded all treatment episodes, where only relatives of the clients were seeking support. We further excluded cases for which gender was documented as “other” (< 0.1%) to allow for propensity matching of the dataset.

For the aim of our analysis, we defined two groups: refugees and German-born individuals as controls. To operationalize the refugee group, we selected all cases with own immigration background from the countries of Afghanistan, Syria and Iraq. Official statistics on the origin of refugees in Germany suggest that the large majority of people immigrating from Afghanistan, Syria and Iraq are asylum seekers [26]. Accordingly, German-born patients formed the comparison group.

We used propensity score matching to pair the two groups. This approach is defined as a quasi-experimental method in which the researcher uses statistical techniques to construct an artificial control group by matching each treated unit with a non-treated unit of similar characteristics and hereby enhances the analytic and interpretive potential of observational (nonrandomized) data [27]. This matching technique facilitates comparison of group profiles by accounting for matching demographics that may be associated with group membership [28]. We matched each refugee episode with one similar episode of a German-born client in terms of age, gender, and main substance of abuse. After the matching, the final study sample was reduced to N = 1.432 (N = 719 refugees and N = 713 German-born).

### Data source and variables

The data of the KDS are divided into the following six areas: (1) Personal data; (2) previous history (e.g., previous care/treatment, pathways to care, funding agencies); (3) sociodemographic data (e.g., living situation, children, migration background, school/education, employment situation, residential situation); (4) addiction-related problem areas (e.g., substance related and behavioral addictions, forms of consumption, related socioeconomic problems, other diagnoses); (5) measures and interventions (e.g., measures implemented, cooperation with other services); (6) treatment completion data (e.g., duration and frequency of contact, type of termination, further referral, contact to self-help).

We selected the dichotomous variable “refugee status” as the main independent variable. Out of all available variables, the following set of dependent variables was selected after discussion in the research team. As dependent variables we chose drug-related variables and socioeconomic problematic areas, dichotomized under “yes/no” variables. Regarding socio-economic variables, we chose age, gender, partnership status, education, living situation at beginning of treatment (living independently/provisional housing), having minor children, employment, and the existence of debts. Finally, having experienced violence (sexual violence or other types of violence) or having exerted violence against others was also analyzed.

### Analysis

The sample was described using descriptive statistics. Categorical data are presented as frequencies and percentages, and continuous variables as means and standard deviations. Statistical significance of categorical variables was tested using chi-squared tests; in case of continuous variables, ANOVA were employed. We set the significance level at α = 0.05; therefore, a p-value < 0.05 was considered as statistically significant. To be able to make additional statements, accompanying odds ratios were calculated. We employed propensity score matching using a multivariate logistic regression model consisting of the variables: age, gender, and main drug (i.e., main reason for entering treatment). We utilized 1:1 nearest neighbor matching. Due to the large proportion of German-born individuals in the initial dataset, we were able to use a very low match tolerance (caliper width) of 0.000003. Statistical analysis was performed using SPSS 26, extended by the Python Essentials plug-in and the FUZZY extension [29].

## Results

### Sociodemographic characteristics

The final sample of refugees was composed of n = 384 (26.8%) cases from Afghanistan, n = 214 (14.9%) from Syria and n = 121 (8.4%) from Iraq (Table 1). Most of the refugee patients were men (n = 699 [97,2%]) and young (mean 27.75 [SD = 10.66]), with half of the refugee sample being less than 25 years old (50.0% (n = 360). In comparison, the original german-born group hat a lower percentage of men (n = 22,682, [71,1%]) and a slight older median age (mean 29.2 SD [8.5]).

**Table 1:** Sociodemographic characteristics and substance use in refugees vs. German-born clients

	Refugees	Germany-born	Total	p
	% (N)	% (N)	% (N)	
Gender				
Man	97.2 (699)	96.8 (690)	97.0 (1389)	0.62
Woman	2.8 (20)	3.2 (23)	3.0 (43)	
Age (mean; SD)	27.75 (10.66)	27.55 (10.06)	27.65 (10.36)	0.71
Main current problematic drug				
Alcohol	17.2 (106)	17.2 (105)	17.2 (211)	0.96
Opioids	20.1 (124)	20.3 (124)	20.2 (248)	
Cannabis	44.8 (276)	45.1 (275)	44.9 (551)	
Sedatives	0.6 (4)	0.7 (4)	0.7 (8)	
Stimulants*	9.1 (56)	9.5 (58)	9.3 (114)	
Tobacco	1.1 (7)	1.0 (6)	1.1 (13)	
Other	0.6 (4)	0.2 (1)	0.4 (5)	
Polysubstance use	6.3 (39)	6.1 (37)	6.2 (76)	
Stable partnership				
No	69.7 (371)	68.7 (395)	69.2 (766)	0.71
Yes	30.3 (161)	31.3 (180)	30.8 (341)	
Basic education				
Basic education	18.0 (83)	10.5 (60)	13.8 (143)	0.00
No basic education	37.0 (171)	13.1 (75)	23.8 (246)	
Primary school	24.5 (113)	32.6 (187)	29.0 (300)	
Secondary school	8.0 (37)	29.5 (169)	19.9 (206)	
Other	12.5 (58)	14.3 (82)	13.5 (140)	
Higher education				
No higher education	78.8 (372)	54.9 (311)	65.8 (683)	0.00
Professional training	19.4 (92)	43.3 (245)	32.4 (337)	
University degree or higher	1.7 (8)	1.8 (10)	1.7 (18)	
Living situation (at beginning of treatment)				
Individual housing	44.0 (262)	50.8 (300)	47.4 (562)	0.02
Provisional housing	56.0 (333)	49.2 (291)	52. (624)	
Minor children				
No	51.3 (369)	65.2 (465)	58.2(834)	< 0.05
Yes	48.7 (350)	34.8 (248)	41.8 (598)	
Employment				
Student	16.5 (92)	24.9 (145)	20.8(237)	< 0.05
Employed	18.8 (105)	26.2 (153)	22.6 (258)	
Unemployed	64.0 (358)	44.9 (262)	54.3 (620)	
Other	0.7 (4)	3.9 (23)	2.4 (27)	
Debts				
No	65.2 (210)	63.5 (257)	64.2 (467)	0.64
Yes	34.8 (112)	36.5 (148)	35.8 (727)	

*In the KDS, amphetamines and cocaine are assessed together under the category “stimulants”

After propensity score matching, the groups did not differ in terms of gender (n = 20 [2.8%] refugee women, vs. n = 23 [3.2%] German-born women; p = 0.62) nor age (mean 27.75 [SD = 10.66] in refugees vs. mean 27.55 [SD = 10.06] in Germany-born; p = 0.71). Half of the refugee sample was less than 25 years old (50.1%).

With respect to sociodemographic variables, groups differed regarding the educational level, the living situation, the fact of having minors in charge and the employment status (p < 0.05). A higher proportion of refugees than Germans lived in provisional housing (n = 333 [56.0%] vs. n = 291 [49.2%]; p = 0.02), were unemployed (n = 358 [64.0%] vs. n = 262 [44.9%]; p < 0.001) and were in charge of minor children (n = 350 [48.7%] vs. n = 248[34.8]; p < 0.05).

### Outcome variables

#### Substance use

Among refugees, the main problematic substance of use at the time of consultation was cannabis (n = 276, 44.8%) followed by opioids (n = 124, 20.1%). In the non-matched German-born group, the most common substance for consultation was alcohol (n = 5652, [43,2%]. In refugees, the most frequent past problematic use also was cannabis (n = 359, 58.6%), followed by alcohol (n = 194, 31.6%) and stimulants (n = 190, 31.0%). The proportions of past problematic drug use differed between the two groups, although it did not reach statistical significance (p = 0.06, Table 2).

**Table 2:** Drug use characteristics, socioeconomic problems, and violence in refugees vs. German-born clients

Problem area			Refugees	German-born	Total	p
			% (N)	% (N)	% (N)	
Drugs	Problematic drug use (multiple responses possible)*	Alcohol	31.6 (194)	28.1 (170)	29.9 (364)	0.06
		Opioids	27.6 (169)	23.9 (145)	25.8 (314)	
		Cannabinoids	58.6 (359)	61.4 (372)	60.0 (731)	
		Sedatives	2.6 (16)	4.5 (27)	3.5 (43)	
		Inhalants	0.3 (2)	0.2 (1)	0.2 (3)	
		Stimulants**	31.0 (190)	28.4 (172)	29.7 (362)	
		Tobacco	10.4 (64)	11.2 (68)	10.8 (132)	
		Others	0.5 (3)	0.2 (1)	0.2 (4)	
	Intravenous drug use	No	91.8 (293)	85.8 (315)	88.6 (608)	0.01
		Yes	8.2 (26)	14.2 (52)	11.4 (78)	
	Needle Sharing	No	100 (111)	78.4 (29)	94.6 (140)	< 0.01
		Yes	0.0 (0)	21.6 (8)	5.4 (8)	
Comorbidities	Impaired physical health	No	43.0 (142)	50.2 (165)	46.6 (307)	0.07
		Yes	57.0 (188)	49.8 (164)	53.4 (352)	
	Impaired mental health	No	17.4 (67)	24.8 (101)	21.2 (168)	0.01
		Yes	82.6 (318)	75.2 (306)	78.8 (624)	
Other problems	Family problems	No	27.5 (92)	34.2 (126)	31.0 (218)	0.05
		Yes	72.5 (243)	65.8 (242)	69.0 (485)	
	Occupational or job problems	No	27.6 (92)	34.1 (126)	31.0 (218)	0.06
		Yes	72.4 (241)	65.9 (244)	69.0 (485)	
	Difficulties with leisure activities	No	36.7 (102)	51.4 (148)	44.2 (250)	< 0.01
		Yes	63.3 (176)	48.6 (140)	55.8 (316)	
	Day organization	No	33.3 (104)	49.5 (146)	41.2 (250)	< 0.01
		Yes	66.7 (208)	50.5 (149)	58.8 (357)	
	Financial situation	No	31.7 (99)	47.3 (159)	39.8 (258)	< 0.01
		Yes	68.3 (213)	52.7 (177)	60.2 (390)	
	Housing	No	39.2 (125)	51.8 (177)	45.7 (302)	< 0.01
		Yes	60.8 (194)	48.2 (165)	54.3 (359)	
	Legal problems	No	36.4 (144)	49.1 (170)	42.3 (314)	< 0.01
		Yes	63.6 (252)	50.9 (176)	57.7 (428)	
Violence	Experiences of sexual abuse	No	97.7 (171)	96.8 (183)	97.3 (354)	0.604
		Yes	2.3 (4)	3.2 (6)	2.7 (10)	
	Other experiences of violence	No	77.1 (148)	87.4 (174)	82.4 (322)	< 0.01
		Yes	22.9 (44)	12.6 (25)	17.6 (69)	
	Violent Behavior	No	82.6 (161)	86.3 (177)	84.5 (338)	0.29
		Yes	17.4 (34)	13.7 (28)	15.5 (62)	

*Problematic use defined by the responsible counselor

**In the KDS, amphetamines and cocaine are assessed together under the category “stimulants”

After propensity score matching, German-born individuals were more likely to have used intravenous drugs than refugees (n = 52 [14.2%] vs. n = 26 [8.2%]; p < 0.05). In subgroup analyses, all three refugee subgroups showed lower odds of intravenous drug use, although the differences did not reach statistical significance (Table 3). None of the individuals in the refugee group reported sharing needles, in contrast to 21.8% of the German-born drug users (p < 0.001).

**Table 3:** Problem areas in the different subgroups of refugees as compared to German-born clients

Problem area		AFG			SYR			IRQ				
		% (N)	OR (95% CI)	p	% (N)	OR (95% CI)	p	% (N)	OR (95% CI)			p
Drug-related*	Intravenous drug use	9.2 (17)	0.61 (0.34–1.10)	0.09	8.0 (7)	0.52 (0.23–1.00)	0.01	4.3 (2)	0.27 (0.01–1.14)			0.06
	Needle sharing	0.0 (0)	0.78 (0.66–0.92)	0.00	0.0 (0)	0.78 (0.66–0.92)	0.08	0.0 (0)	0.78 (0.66–0.92)			0.11
Comorbidities	Physical	61.8 (115)	1.62 (1.13–2.35)	0.01	43.3 (43)	0.77 (0.49–1.21)	0.26	66.7 (30)	2.01 (1.04–3.87)			0.03
	Mental	86.5 (180)	2.12 (1.34–3.35)	< 0.01	77.9 (122)	1.16 (0.71–1.88)	0.54	78.2 (43)	1.18 (0.60–2.33)			0.63
Other problems	Family situation	77.6 (142)	1.80 (1.19–2.71)	0.05	64.4 (67)	0.94 (0.59–1.48)	0.80	70.8 (34)	1.26 (0.65–2.44)			0.48
	Debts	37.0 (60)	1.02 (0.70–1.49)	0.91	30.1 (72)	0.74 (0.46–1.19)	0.22	36.8 (21)	1.01 (0.57–1.79)			0.97
	Temporary housing	63.6 (208)	1.81 (1.36–2.37)	< 0.01	43.9 (75)	0.80 (0.57–1.05)	0.21	51.5 (50)	1.09 (0.71–1.68)			0.67
	Occupational situation	75.0 (138)	1.55 (1.04–2.30)	0.03	67.6 (71)	1.08 (0.67–1.71)	0.75	72.7 (32)	1.37 (0.68–2.76)			0.37
	Free Time	70.9 (107)	2.57 (1.69–3.90)	< 0.01	53.3 (48)	1.21 (0.75–1.94)	0.43	56.8 (21)	1.39 (0.69–2.77)			0.35
	Daily structure	69.3 (113)	2.21 (1.48–3.31)	< 0.01	63.8 (67)	1.73 (1.09–2.73)	0.02	63.6 (28)	1.71 (0.89–3.30)			0.10
	Financial situation	70.8 (121)	2.17 (1.47–3.21)	< 0.01	64.6 (64)	1.64 (1.03–2.61)	0.03	66.7 (28)	1.79 (0.91–3.53)			0.08
	Legal situation	59.2 (129)	1.40 (0.99–1.97)	0.05	66.4 (77)	1.90 (1.23–2.96)	< 0.01	74.2 (46)	2.78 (1.51–5.10)			< 0.01
Violence	Sexual Abuse	1.0 (1)	0.32 (0.04–2.70)	0.27	3.8 (2)	1.22 (0.24–6.21)	0.81	3.7 (1)		1.17(0.13–10.10)	0.88	
	Other types of violence	22.9 (24)	2.06 (1.11–3.83)	0.02	22.4 (13)	2.01 (0.95–4.24)	0.06	24.1 (7)		2.21 (0.86–5.71)	0.09	
	Violent behavior	13.2 (4)	0.96 (0.48–1.91)	0.91	20.0 (12)	1.58 (0.75–3.33)	0.23	27.6 (8)		2.40 (0.97–5.95)	0.05	

*Types of substance use not included as it was used for propensity matching of client

#### Comorbidities

After propensity score matching, the proportion of impaired physical health was higher in refugees, although the differences did not reach statistical significance (57.0% vs. 49.8%; p = 0.07). In the subgroup analyses, people from Afghanistan and Iraq were more likely to suffer from physical comorbidities (61.8%, OR = 1.6 [1.13–2.35], p = 0.01 and 66.7%, OR = 2.01 [1.04–3.87], p = 0.03, respectively), which was not the case for refugees from Syria (43.3%, OR = 0.77 [0.49–1.21]; p = 0.26). Mental health problems were also more frequent in refugees (82.6 vs. 75.2%, p = 0.01), and subgroup analyses showed that namely refuges from Afghanistan accounted for this difference (86.5%, OR = 2.12 [1.34–3.35]; p = 0.001).

#### Socio-economic areas

Problems in the areas of family, finances, legal situation, housing, day organization and leisure activities were all significantly higher in refugees compared to German-born individuals (Table 2). In subgroup analyses, clients from Afghanistan and Syria were more likely to report issues with structuring their day (69.3%, OR = 2.21 [1.48–3.31], p < 0.01 and 63.8%, OR = 1.73 [1.09 -2.73], p = 0.02, respectively); their financial situation (70.8%, OR = 2.17 [1.47–3.21], p < 0.01 and 64.6%, OR = 1.64 [1.03–2.61], p = 0.03, respectively). All subgroups of refugees showed significand higher proportion of problems respecting the legal situation. In the subgroup of people from Afghanistan, the odds of temporary housing (63.6%, OR = 1.81 [1.36–2.37], p < 0.001) and problems with occupation/employment (75.0%, OR = 1.55 [1.04–2.30], p = 0.03) were significantly higher than in the German-born control group. Debts were not significant different between groups, with an overall prevalence of 35.8% (Tables 2 and 3).

#### Violence

Experiences of violence other than sexual abuse were more frequent in the refugee population (22.9% vs. 12.6%, p = 0.007), and reached significance in the subgroup of people from Afghanistan (22.9%, OR = 2.06 [1.11–3.83], p = 0.02). Exertion of violence was not significantly different between refugees and German-born individuals (17.4% vs. 13.7%, p = 0.24), and neither in any of the refugee subgroups as compared to German-born controls (Table 3). History of sexual abuse was not significantly different, with an overall prevalence of 2.7% (n = 10) (Table 2). In the subgroup analysis, sexual abuse did not differ in any subgroup with respect to the German-born controls.

## Discussion

In our study, we compared the clinical profile of people with refugee background with a German-born comparison group, in order to describe differences or similarities. Our findings provide important insights into the needs of this understudied population and could be useful in the development of programs tailored to the needs of this target group.

Our first important finding is the overrepresentation of men in our sample. In 2020, about 40% of the refugees in Germany were women [30], in contrast to only 2.8% found in our treatment-seeking sample. As a general trend, the prevalence of substance use disorders is higher in men than in women [31, 32]. In the particular case of refugees, other studies also showed that being a male refugee was a risk factor for alcohol consumption [14, 16, 33–35]. A study of refugees resettled in the United States [34] found that the prevalence of hazardous drinking was 5.1% in men as compared to 0.6% in women. However, the prevalence of substance use disorders in a study from Uganda did not differ with respect to gender [20]. The stigmatization of women with substance use disorders may be a reason why the proportion of refugee women was minimal in our sample. It has also been shown that there are gender-related barriers to treatment. Women enter treatment later than men and when they do, they usually have more severe symptomatology and additional psychosocial stressors such as trauma history and marginalization [36, 37]. For instance, a qualitative study from the Mae La refugee camp in Thailand, gender-related dimensions of substance abuse emerged in the thematic analysis. Individuals reported that women are supposed to exert more self-control and do not conduct behaviors that contradict the social norms [14]. It is possible that this will also apply to our sample and more generally to female populations.

We found significant differences between both groups regarding the pattern of drug use. In the German general population, alcohol is the most commonly used substance and, consequently, also ranks first in German treatment services [25, 38], and this was also true in our German non-matched population (43% consulting due to alcohol use). However, in our refugee sample, cannabis and opioids were the main problematic drugs. Other studies among refugee populations revealed different results. For example, in a study of Syrian refugees in Lebanese camps, the use of alcohol was higher than cannabis, although the cannabis consumption was also large, reaching a prevalence of 30%. Iraqi refugees resettled in the United States also reported a higher prevalence of alcohol abuse than abuse of other substances (1.9 vs 0.6, respectively) [39]. However, samples may not be comparable, as we analyzed treatment seeking individuals and not refugees from the general population. Additionally, the discordancy in results may be caused by population heterogeneity, differences in host countries and the duration of the resettlement [12]. Still, cannabis use seems to be consistently high in displaced populations and should be a target for future prevention programs.

Injection drug use patterns among people from culturally diverse backgrounds have been barely studied so far. We found that the prevalence of intravenous drug use was almost twice as high in the German-born population, and no refugee reported needle sharing. These results may be explained by a combination of social and cultural factors. In a qualitative study in Australia, for instance, African refugees were interviewed about their perceptions of intravenous drug use, and they perceived it as unnatural, risky and immoral. Although intravenous drugs were widely available, individuals engaged in several practices to ensure that the family did not find out about their intravenous use [40]. For this reason, we cannot discard the possibility of reporting bias, as the stigma related to intravenous drug use may result in underreporting of this practice. If this is the case, we may need special efforts to reach the population of refugees who are using intravenous drugs.

Medical and psychiatric associated problems were more prevalent in the refugee group and reached significance in the Afghan and Iraqi subgroups compared to the German-born counterparts. A high burden of mental health problems in refugee populations has consistently been reported both in international [41] and German samples [42]. This underscores the need of integrating appropriate interventions to address these problems in substance use settings [43], as refugee populations frequently present additional medical comorbidities. Research on the physical health of refugees in Europe is scarce, but some studies point out to a high prevalence of communicable diseases, mainly due to poor living conditions [44].

To sum up, not only they have more comorbidity, but also face numerous barriers to access healthcare, what prevents them to an adequate management of their disorders. Several obstacles have been highlighted, such as linguistic barriers, lack of cultural mediation, bureaucratic and administrative barriers, or low health literacy [21, 41, 45] Our sample showed that refugees usually presented a lower education level, a factor that may also influence their health literacy and access to the health system.

In line with previous studies, refugees presented significant difficulties in social areas such as dealing with temporary housing, lack of free time organization and employment. Importantly, all of these problems were risk factors for substance abuse in previous studies [46], as the literature consistently suggests a relation between postmigration stressors and psychiatric outcomes independent of the country of origin or destination [15, 16, 20, 33]. Although we cannot make causal inferences, it is possible that these socio-structural difficulties contribute to substance use and abuse in refugees. For example, the lack of stable housing may marginalize individuals and increase their exposure to drugs. Additionally, it may create feelings of stress and hopelessness, with substance use as a coping mechanism [18, 47]. As Bogic et al. showed [35], postmigration difficulties can interfere with the development of a SUD. In their study, differences in the prevalence of SUD between countries were fully explained by differences in postmigration factors, being temporary residency one of the main explanatory variable. Another important postmigration factor is the lack of day organization in refugee populations, which also may precipitate the use of drugs [33]. For example, among Bhutanese refugees in Nepal, the main reasons for alcohol consumption were unemployment, lack of daily structure and lack of free time activities. In our study, we also found that refugees were more likely to present difficulties in day structure than German-born individuals. Given the potential importance of social factors for substance use among refugees, it has been suggested to expand the focus on this problem beyond individual to the social ecological context in any attempt, including prevention, treatment and research [47].

In our sample, refugees presented a significantly higher proportion of legal problems compared to German-born individuals. It can be assumed that some of them may be related to the asylum process. According to previous studies, an unclear asylum status is related to a higher risk of psychopathology [48]. Therefore, we consider that improving prospects and opportunities of refugee populations could help to diminish their risk of substance use [47]. Legal problems could also be related to violence or misbehavior. However, we found that violent behavior was more common in German-born individuals, while refugees were more likely to have been victims of violence. The level of education significantly differed between the groups. Refugees showed lower education levels than the German counterparts, which could compromise their health literacy with respect to the available addiction treatments.

Finally, we compared the history of sexual abuse between both groups, and did not find statistical significant differences. Interestingly, these numbers were similar to those reported in the general German population [49]. Other studies, however, point to a higher prevalence of sexual abuse in refugees. In a sample of refugee children in the US, 18.2% reported having been sexually assaulted and 7.3% having been sexually maltreated/abused, numbers much higher than in our sample. It is possible that the stigma related to sexual abuse interferes with the ability of the client to open up about it. It is also possible that healthcare workers are not addressing this topic, or that they are not enough trained to facilitate the disclosure of the sexual abuse, particularly in people with different cultural backgrounds.

The main limitations of our study are the cross-sectional design and some missing data. Clinical information is only saved in the KDS if addressed in the consultation, and therefore the sample size of the different items is varying. Also, there is a risk of reporting bias. Other cultures may have different cultural understandings of mental health may influence the topics approached in treatment. Some topics, such as sexual abuse, may be even more stigmatized and underreported than in the German culture. The main strength of our study is the sample size. The number of episodes allowed the matching of every refugee episode with one without migration background, enabling a better comparison between groups. This allows us to shed a light on particular areas in which refugees may need psychosocial interventions.

## Conclusions

In conclusion, our findings suggest that refugees with substance use disorders show a wide spectrum of needs compared to Germany-born individuals. They are subject to significant post migration difficulties as well problems with their mental and physical health. In our study, refugees had a high burden of social problems such as temporary housing, difficulties with day structure, and being victims of violence. These post-migration difficulties may influence the use of substances. For this reason, more research is needed to determine how these social factors influence substance use, in order to improve the social contexts of refugees in receiving countries and develop prevention and intervention programs to address this populations. Our findings highlight the need to link addiction treatment with other parts of the health care and legal systems, in order to meet the complex needs of this vulnerable population.

## Data Availability

No datasets were generated or analysed during the current study.

## Footnotes

1 the term refugees used throughout the study and the article refers to the common parlance definition of the term, not a legal definition; refugees comprise different groups of forcibly displaces populations, including forced migrants, undocumented migrants, asylum seekers, and recognized refugees as they share the commonality of ‘perceived forcedness’ to emigrate.
